# Ethanol and NaCl-Induced Gold Nanoparticle Aggregation Toxicity toward DNA Investigated with a DNA/GCE Biosensor

**DOI:** 10.3390/s23073425

**Published:** 2023-03-24

**Authors:** Jana Blaškovičová, Vlastimil Vyskočil, Michal Augustín, Andrea Purdešová

**Affiliations:** 1 Institute of Analytical Chemistry, Faculty of Chemical and Food Technology, Slovak University of Technology, Radlinského 9, 812 37 Bratislava, Slovakia; 2 UNESCO Laboratory of Environmental Electrochemistry, Department of Analytical Chemistry, Faculty of Science, Charles University, Hlavova 2030/8, 128 43 Prague, Czech Republic; 3 Department of Chemistry, Faculty of Natural Sciences, University of Ss. Cyril and Methodius in Trnava, Nám. J. Herdu 2, 917 01 Trnava, Slovakia

**Keywords:** aggregation, DNA/GCE biosensor, gold nanoparticles, toxicity, ethanol, NaCl

## Abstract

Engineered nanomaterials are becoming increasingly common in commercial and consumer products and pose a serious toxicological threat. Exposure of human organisms to nanomaterials can occur by inhalation, oral intake, or dermal transport. Together with the consumption of alcohol in the physiological environment of the body containing NaCl, this has raised concerns about the potentially harmful effects of ingested nanomaterials on human health. Although gold nanoparticles (AuNPs) exhibit great potential for various biomedical applications, there is some inconsistency in the case of the unambiguous genotoxicity of AuNPs due to differences in their shape, size, solubility, and exposure time. A DNA/GCE (DNA/glassy carbon electrode) biosensor was used to study ethanol (EtOH) and NaCl-induced gold nanoparticle aggregation genotoxicity under UV light in this study. The genotoxic effect of dispersed and aggregated negatively charged gold nanoparticles AuNP1 (8 nm) and AuNP2 (30 nm) toward salmon sperm double-stranded dsDNA was monitored by cyclic and square-wave voltammetry (CV, SWV). Electrochemical impedance spectroscopy (EIS) was used for a surface study of the biosensor. The aggregation of AuNPs was monitored by UV-vis spectroscopy. AuNP1 aggregates formed by 30% *v/v* EtOH and 0.15 mol·L^−1^ NaCl caused the greatest damage to the biosensor DNA layer.

## 1. Introduction

Exposure of organisms to engineered nanomaterials causes concerns for human health. The growing presence of various nanoparticles in the environment, cosmetics, food, and pharmaceutical products poses a serious toxicological threat. Nanoparticles can enter the human body in different ways, the most common of which are inhalation, oral intake, and skin absorption [1]. The toxicity of engineered nanoparticles depends on size, shape, and surface modification. In addition, the environment in which they exist is of interest [2].

Gold nanoparticles (AuNPs) are widely used in bio-nanotechnology due to their biocompatibility and low toxicity [3]. AuNPs are easy to prepare with good size and shape control, as well as unique optical properties and functionalization [4,5]. In many areas, the use of gold nanoparticles is increasing. The presence of AuNPs in polymer composites plays a key role in food packaging [6,7,8]. AuNPs are used in wound healing [9,10], other diagnostic and therapeutic medical applications [11,12,13], and cosmetics [14,15,16]. The increased exposure of the human body to AuNPs raises concerns about the safety of such nanoparticles.

Together with the use of AuNPs, there is simultaneous consumption or exposure to ethanol in foodstuffs, medications, and cosmetics. Ethanol is often an active ingredient in oral, parenteral, and topical drugs [17]. Ethanol has been used in a variety of cases for injection therapy of trigeminal neuralgia [18], tumor therapy [19,20,21,22], and as a form of pain relief [23,24]. Ethanol is absorbed by passive alcoholization by hand rubbing [25]. Despite the high levels of ethanol consumption worldwide, inexpensive parapharmaceuticals based on different ethanol concentrations (such as disinfectants and deodorizing solutions) are also consumed by the poor population [26].

Nevertheless, the transfer of AuNPs from an aqueous system to physiological media causes instability, loss of the original optical plasmonic band, aggregation, or precipitation [2]. One of the reasons for these phenomena is that physiological media contain salt, which may cause nanoparticle aggregation [27]. Due to their small dimensions, AuNPs can enter cell structures and interact with DNA [28]. Likewise, the interaction between AuNPs and DNA depends on the salt concentration [27] and DNA structure [5]. The strength of the hydrophobic and electrostatic forces of AuNPs/DNA were reported to be remarkably modified by the presence of alcohols in media [29]. In addition, for lower ethanol concentrations of up to 30% *v*/*v*, DNA exists in the B form, whereas the conformational change of DNA to the C form occurs at higher ethanol concentrations of up to 65% due to the shrinkage of the double helix [29,30,31].

Therefore, it is interesting to observe whether the aggregation of nanoparticles under the influence of salt in a physiological medium and different concentrations of ethanol affects DNA damage.

Many electrochemical biosensors containing DNA structures, such as dsDNA, ssDNA (single-stranded DNA), aptamers, oligonucleotides, and hairpins, detect various analytes and pathogens [32,33], but few studies have been devoted to monitoring the toxicity of these analytes in the DNA structure. An electrochemical DNA biosensor was used for detection of carcinogenicity [34]. In another study, Machini et al. used dsDNA electrochemical biosensors to monitor the drug metformin–dsDNA interaction based on changes in the oxidation peak current of DNA base residues. They identified minor groove binding and unwinding of the DNA structure after prolonged exposure [35]. Furthermore, DNA biosensors were used to observe the toxicity of drugs in DNA structure and to assess their interaction [36,37,38]. Many DNA biosensors have been used to monitor the antioxidant properties of foodstuffs [39]. The authors of [40] described the antioxidant properties of algae detected by decreased DNA damage using a carbon screen-printed electrode biosensor with a surface-attached DNA sequence as a result of protection against Fenton reaction products.

A DNA/GCE biosensor and its UV irradiation were selected for a detailed study of damage to surface-attached DNA structures. This approach has been used in several studies to monitor the toxicity of drugs [41,42], nanoparticles [43,44], and antioxidants [45] in DNA structures. In this procedure, it is possible to study the damage or the protective effect of selected analytes. A DNA/GCE biosensor was selected as a powerful tool to investigate the DNA damage caused by AuNPs under experimental conditions in combination with a physiological concentration of NaCl (0.15 mol·L^−1^) and various ethanol concentrations. The objective of this study was to evaluate the toxicity of gold nanoparticle aggregation induced by ethanol and NaCl toward dsDNA. A DNA/GCE biosensor was selected as a massive, easy-to-prepare, inexpensive, and specific tool for this experiment. It was interesting to observe whether the interaction of AuNP aggregates created in the presence of ethanol and NaCl with surface-attached DNA was strong enough to protect the DNA layer or whether the presence of AuNP aggregates would contribute to increased DNA damage under simultaneous UV irradiation.

## 2. Materials and Methods

### 2.1. Materials

Gold nanoparticles were synthesized at the Institute of Analytical Chemistry, Faculty of Chemical and Food Technology, Slovak University of Technology in Bratislava (Slovakia). Low-molecular-weight salmon sperm dsDNA was purchased from Sigma-Aldrich (Germany). Na_2_HPO_4_, NaH_2_PO_4_, K_3_[Fe(CN)_6_], and K_4_[Fe(CN)_6_]·3 H_2_O were obtained from Lachema Brno (Czech Republic). CH_3_CH_2_OH and NaCl were provided by Centralchem Bratislava (Slovakia). Other chemicals were obtained from Mikrochem (Slovakia) and Lachema (Czech Republic). All chemicals were of analytical reagent-grade purity. Nanopure water with resistivity above 18 MΩ·cm (Millipore Milli-Q system) was used for all experiments.

### 2.2. Synthesis of Gold Nanoparticles

All glassware used in the following procedure was cleaned in a bath of freshly prepared 3:1 HNO_3_–HCl and rinsed thoroughly in twice-distilled water. A 1% (*w*/*w*) solution of HAuCl_4_ and trisodium citrate was filtered through a 22 μm microporous membrane filter before use. Two types of AuNPs were chemically synthesized, namely AuNP1 and AuNP2. Synthesis of trisodium citrate dihydrate-capped AuNP2 was based on simple wet chemical reduction of chloroauric ions with slight modification of the protocol proposed by Turkevich. Trisodium citrate dihydrate was used as both a reducing and stabilizing agent. Briefly, 250 mL of 1.0 mmol·L^−1^ HAuCl_4_ was boiled while stirring in a 500 mL round-bottom flask. Then, 25 mL of 38.8 mmol·L^−1^ sodium citrate was quickly added to the solution. The resulting mixture was refluxed for 30 min with continuous stirring. The flask was allowed cool to room temperature and was stored in the dark at 4 °C until use. The size of nanoparticles characterized by standard scanning electron microscopy (SEM) (solution sputtered on a solid silicon substrate) was 30 nm. AuNP1s of 8–12 nm were synthesized by reduction of gold (III) chloride trihydrate by sodium borohydride. Then, 4% HAuCl_4_ solution was mixed with 0.2 mol·L^−1^ K_2_CO_3_ in 100 mL nanopure water on ice for 30 min. Fresh 0.5 mg·mL^−1^ NaBH_4_ was added to the abovementioned mixture under rapid stirring until the color changed from blue–purple to red–orange. Nanoparticles were stored in the dark at 4 °C for further use.

### 2.3. Apparatus

A glassy carbon working electrode (GCE, Metrohm, The Netherlands) with a disc diameter of 3 mm, a Ag/AgCl/3 mol·L^−1^ KCl reference electrode, and a platinum wire counter electrode (L-CHEM, Czech Republic) formed a three-electrode system used in all electrochemical experiments. For voltammetric and impedimetric measurements, an Autolab PGSTAT12 potentiostat/galvanostat electrochemical system (Metrohm, The Netherlands) driven by NOVA version 1.10.23 (Metrohm, The Netherlands) was used. All measurements were performed in 20 mL glass cells at ambient temperature. UV irradiation was applied with an OSRAM PURITEC HNS S 9 W UV lamp with a wavelength of 253.7 nm and a radiation power of 2.5 W (Gajdoš, Slovakia). UV-vis absorption spectra were obtained with an Evolution 200 Series UV-vis spectrophotometer (Thermo Fisher Scientific, Waltham, MA, USA) in the wavelength range of 280 to 800 nm in 12.5 × 12.5 × 45 mm semi-micro UV cuvettes (Brand, Germany) under the following conditions: bandwidth of 2 nm, 0.25 s integration time, 1 nm data interval, and scan speed of 80 nm·s^−1^. Instrument control and data processing were performed using Thermo INSIGHT software version 1.4.

### 2.4. Preparation of the Biosensor

The GCE surface was mechanically cleaned with a polishing cloth (BUEHLER, UK) and 0.3 μm alumina suspension (Metrohm, The Netherlands). Electrochemical cleaning and GCE pretreatment at a potential of 1.6 V for 300 s in 0.1 mol·L^−1^ phosphate buffer (PB) solution of pH 7.4 were used for electrode polarization. To stabilize the CV response of the working electrode, 15 scans were performed in 1 × 10^−3^ mol·L^−1^ [Fe(CN)_6_]^3−/4−^ redox indicator in the potential range of 1.0 to −0.8 V before measurement. A salmon sperm dsDNA stock solution was prepared by dissolving the DNA in nanopure water to a concentration of 1 mg·mL^−1^. For the preparation of the DNA/GCE biosensor, the pretreated GCE surface was covered with 4 μL of DNA stock solution and dried. The prepared DNA/GCE biosensor was immersed in 0.1 mol·L^−1^ PB (pH 7.4) solution for 2 min before use.

### 2.5. Biosensor Treatment

For treatment of the DNA/GCE biosensor with AuNPs, a solution of 4 μL of AuNPs diluted in 0.1 mol·L^−1^ PB (pH 7.4) (1:20 *v*/*v*) was dropped on the surface of the DNA/GCE biosensor and incubated at ambient temperature for 30 min. For the treatment of the DNA/GCE biosensor with EtOH and NaCl, 4 μL of EtOH-NaCl solution diluted in 0.1 mol·L^−1^ PB (pH 7.4) was dropped on the surface of the DNA/GCE biosensor and incubated at ambient temperature for 30 min. The EtOH-NaCl solution was prepared by mixing 1 mol·L^−1^ NaCl, 96% (*v/v*) EtOH, and 0.1 mol·L^−1^ PB (pH 7.4) to a final concentration of NaCl corresponding to 0.15 mol·L^−1^. The final concentration of EtOH was 30% (*v*/*v*), 40% (*v*/*v*), or 50% (*v*/*v*) according to the experimental setup. To determine the effect of AuNPs in the presence of EtOH and NaCl on the surface-attached DNA, AuNP1/AuNP2 was diluted with 0.1 mol·L^−1^ PB (pH 7.4) containing 0.15 mol·L^−1^ NaCl and 30% (*v*/*v*), 40% (*v*/*v*), or 50% (*v*/*v*) EtOH in a volume ratio 1:20. Then, 4 μL of this solution was dropped onto the surface of the DNA/GCE biosensor and incubated for 30 min at ambient temperature.

### 2.6. Methods (Procedures)

Cyclic voltammetry scans were recorded within the potential range of 0.8 to −0.4 V in 0.1 mol·L^−1^ PB (pH 7.4) containing 1 mmol·L^−1^ [Fe(CN)_6_]^3−/4−^ redox indicator at a scan rate of 100 mV·s^−1^ with a potential step of 2 mV. Square-wave voltammograms (SWVs) were obtained at a potential step of 4 mV, a scan rate of 200 mV·s^−1^, a pulse amplitude of 20 mV, and a frequency of 50 Hz. Electrochemical impedance spectroscopy (EIS) was performed in 1 mmol·L^−1^ [Fe(CN)_6_]^3−/4−^ in 0.1 mol·L^−1^ PB (pH 7.4) with a polarization potential of 0.26 V, a frequency range of 0.1 Hz to 5000 Hz (51 frequency steps), and an amplitude of 10 mV. All measurements were performed at a laboratory temperature of 21 °C in a 20 mL electrochemical cell.

### 2.7. Data Treatment

Following CV measurements, the portion of surviving DNA after the UV irradiation was expressed as the normalized biosensor response using the following equation:

(1) $$∆I_{rel}=\frac{I_{survDNA}-I_{GCE}}{I_{DNA}-I_{GCE}}\times 100\%$$

where *I_survDNA_* and *I_DNA_* are the anodic current values for the 1·10^−3^ mol·L^−1^ [Fe(CN)_6_]^3−/4−^ redox indicator obtained using the DNA/GCE biosensor after and before the irradiation, respectively, and *I_GCE_* represents the [Fe(CN)_6_]^3−/4−^ anodic peak current response on the bare GCE.

## 3. Results and Discussion

### 3.1. Spectrophotometric Determination of Gold Nanoparticle Aggregates

**Figure 1:**
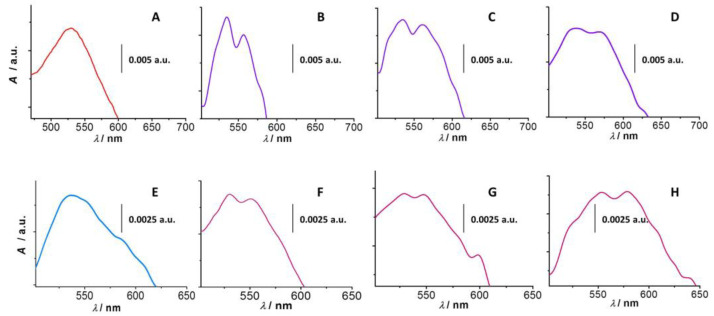
UV-vis spectra of AuNP1 (**A**–**D**) and AuNP2 (**E**,**F**). The absorbance of the dispersed form is shown in panels (**A**) (AuNP1) and (**E**) (AuNP2). AuNP1 in the presence of 0.15 mol·L^−1^ NaCl and 30% *v*/*v* EtOH (**B**), 40% *v*/*v* EtOH (**C**), or 50% *v*/*v* EtOH (**D**) show differences in UV-vis spectra due to their aggregation. Similar differences in absorbance are observed within AuNP2 in the presence of 0.15 mol·L^−1^ NaCl and 30% *v*/*v* EtOH (**F**), 40% *v*/*v* EtOH (**G**), or 50% *v*/*v* EtOH (**H**).

In order to investigate the aggregation of gold nanoparticles in the presence of a physiological concentration of salt and 30, 40, or 50% (*v*/*v*) ethanol, the UV-vis absorption spectra of pure AuNPs and AuNPs after the addition of NaCl/EtOH mixtures were measured. AuNP1 (size of 8 nm) shows an absorption peak maximum at a wavelength of 520 nm (Figure 1A). The addition of EtOH and NaCl caused the formation of a new absorption peak with a maximum at 560 nm, which corresponds to the absorption of radiation by the newly formed gold nanoparticle aggregates. Figure 1B–D show AuNP1 absorption spectra in the presence of 30%, 40%, and 50% *v/v* EtOH in 0.15 mol·L^−1^ NaCl, respectively. It is known that the addition of salt leads to the aggregation of nanoparticles, followed by changes in the plasmonic peak, such as broadening of its width [27]. Furthermore, the addition of EtOH causes a decrease in the dielectric constant of the solution and triggers the effect of the present monovalent sodium cations, which partially destabilizes the negative charge of the nanoparticles and increases their aggregation. A similar but somewhat different phenomenon is observed in the case of AuNP2 with an average particle size of 30 nm, which shows an absorption peak maximum at a wavelength of 540 nm (Figure 1E). A bathochromic (red) shift compared to AuNP1 (520 nm) is present due to the larger size of the nanoparticles. Yahaya et al. showed a similar red UV-vis shift with increasing size of AuNPs [46]. In some cases, the bathochromic shift can also be affected by the ligand exchange [47]. In the case of AuNP2 aggregates formed in the presence of 30% (Figure 1F) and 40% (Figure 1G) EtOH, the hypsochromic (blue) shift of the original absorption peak of the dispersed nanoparticles from 540 to 530 nm and the formation of a new absorption peak at a wavelength of 550 nm belonging to AuNP2 aggregates appear. The hypsochromic shift of AuNPs is most likely due to the changes in the particle coating. In the literature, AuNPs were described to cause a blue shift in the UV-vis spectrum from 518 to 515 nm when uncoated [48]. In our experiment, the exception was the AuNPs clusters, which formed at a 50% ethanol concentration when the absorption peak of the dispersed particles bathochromically shifted from 540 to 550 nm, and a new absorption peak appeared at a wavelength of about 580 nm (Figure 1H). This phenomenon may be caused by a self-aggregation of the gold nanoparticles due to the synergistic effect of NaCl and the high EtOH concentration. Grueso et al. reported similar self-aggregation of gold nanoparticles in the presence of EtOH in the medium at concentrations above 40% *v*/*v* [29]. A change in the original morphology of the nanoparticles and the formation of a new aggregate form are also possible.

### 3.2. Effect of Dispersed AuNPs on the DNA/GCE Biosensor

The next step was to study the effect of dispersed AuNPs on the DNA immobilized on the surface of the DNA/GCE biosensor. The surface of the prepared DNA/GCE biosensor was modified by 4 μL of AuNP1 or AuNP2 diluted in 0.1 mol·L^−1^ PB (pH 7.4) in a ratio of 1:20 without salt addition. The modified AuNP/DNA/GCE biosensor was irradiated with UV-C light for selected time intervals of 20 to 900 s, and damage to the salmon sperm dsDNA was monitored by voltammetric methods (CV, SWV) and EIS.

**Figure 2:**
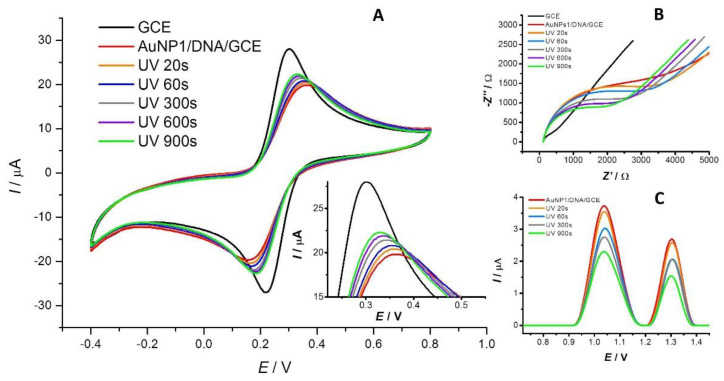
The response of the DNA/GCE biosensor to dispersed AuNP1 was measured by CV (**A**), SWV (**C**), and EIS (**B**) after the 20 s (orange curve), 60 s (blue curve), 300 s (gray curve), 600 s (purple curve), and 900 s (green curve) of UV irradiation. The black color represents the signal of the bare GCE, and the red color represents the response of the DNA/GCE in the presence of AuNP1. CV and EIS show the response of the [Fe(CN)_6_]^3−/4−^ redox indicator, and SWV shows the measured current response for guanine and adenine DNA moieties.

**Figure 3:**
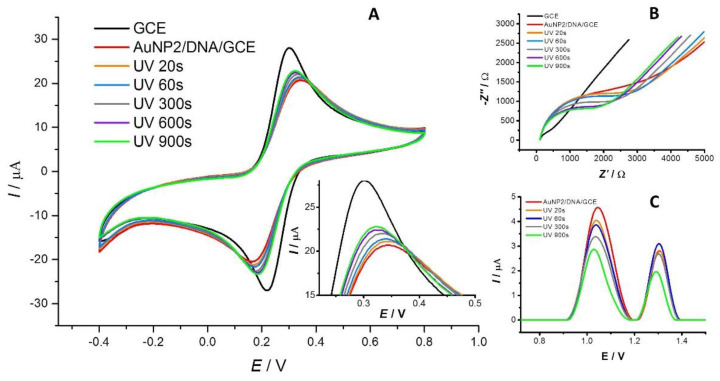
The response of the DNA/GCE biosensor to dispersed AuNP2 was measured by CV (**A**), SWV (**C**), and EIS (**B**) after the 20 s (orange curve), 60 s (blue curve), 300 s (gray curve), 600 s (purple curve), and 900 s (green curve) of UV irradiation. The black color represents the signal of the bare GCE, and the red color represents the response of the DNA/GCE in the presence of AuNP2. CV and EIS show the response of the [Fe(CN)_6_]^3−/4−^ redox indicator, and SWV shows the measured current response for guanine and adenine DNA moieties.

**Figure 4:**
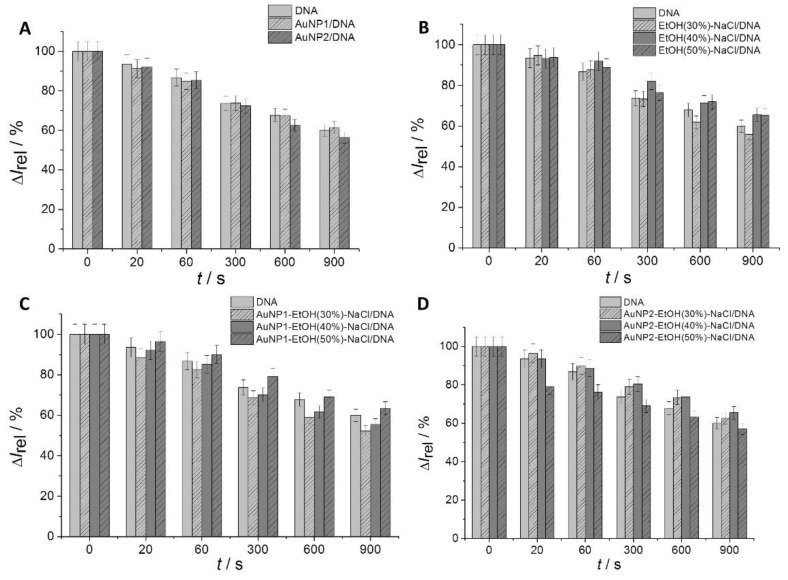
Amount of surviving DNA calculated based on normalized CV responses after treatment of the DNA/GCE with AuNP1 and AuNP2 alone (**A**); after treatment of the DNA/GCE in the presence of 0.15 mol·L^−1^ NaCl and 30%, 40%, or 50% *v*/*v* EtOH (**B**); after treatment of the DNA/GCE in the presence of AuNP1 and 0.15 mol·L^−1^ NaCl and 30%, 40%, or 50% *v*/*v* EtOH (**C**); and after treatment of the DNA/GCE in the presence of AuNP2 and 0.15 mol·L^−1^ NaCl and 30%, 40%, or 50% *v*/*v* EtOH (**D**) under UV irradiation for 0, 20, 60, 300, 600, and 900 s.

Figure 2A and Figure 3A show the effect of AuNP1 and AuNP2 on the DNA attached to the surface of the DNA/GCE biosensor monitored by CV. The black and red curves represent the response of the [Fe(CN)_6_]^3−/4−^ redox indicator on the bare GCE and the DNA/GCE, respectively. The binding of DNA to the surface of the working electrode leads to a decrease in the indicator signal due to electrostatic repulsions between the ions of the redox indicator and the negatively charged sugar–phosphate backbone of DNA. Time-dependent UV-light-induced DNA damage is reflected by an increase in the redox indicator anodic peak current response signal due to better access of the redox indicator to the active surface of the working electrode. The orange, blue, gray, purple, and green lines represent irradiation durations of the 20, 60, 300, 600, and 900 s, respectively. The amount of surviving dsDNA was calculated based on the anodic peak current response signal values of the [Fe(CN)_6_]^3−/4−^ redox indicator according to the Equation (1) mentioned in the Section 2. For surface-attached DNA biomolecules, DNA in the presence of AuNP1, and DNA exposed to AuNP2, the portion of surviving DNA after 20–900 s of exposure to UV irradiation was 93.5–60.0%, 91.3–61.3%, and 92.0–56.6%, respectively (Figure 4A). In the case of dispersed AuNP2, no significant difference in terms of the increase in the CV signal of the redox indicator was observed compared to dispersed AuNP1 during the 20–300 s time interval. This supports the hypothesis that these nanoparticles have a similar effect on the dsDNA layer. After longer periods (600–900 s) of irradiation, a slightly protective effect of AuNP1 and little damage to the dsDNA layer caused by AuNP2 were found (Figure 2A and Figure 3A). These results were confirmed by an agarose gel electrophoresis experiment (data not shown), SWV (Figure 2C and Figure 3C), and EIS (Figure 2B and Figure 3B).

Figure 2C shows the SW voltammograms corresponding to the response of the adenine and guanine residue moieties under the simultaneous action of AuNP1 and UV light irradiation during the 20–900 s time interval. DNA damage was detected as early as 20 s (orange curve), as manifested by a decrease in the current response signal of guanine moieties at a potential value of 1.04 V and adenine moieties at 1.32 V. As the irradiation time increased (60–300 s), the signal decreased further. The current response of DNA base residues remained voltage-stable, indicating a negligible interaction between dAuNP1 and dsDNA. The SW voltammograms corresponding to the response of the adenine and guanine residues under the simultaneous action of AuNP2 and UV-C radiation in the 20–900 s time interval (Figure 3C) show practically the same trend of decreasing signals of DNA base residues, which are voltage-stable, as in the case of AuNP1.

Similar results were obtained in the EIS experiments (Figure 2B and Figure 3B). The black curve represents the bare GCE signal. The attachment of DNA to the surface of the working electrode (red curve) resulted in an extension of the semicircle in the Nyquist plot. Subsequent DNA damage (orange to green curve) appears as a shrinking of the semicircle in the Nyquist diagram, resulting from a decrease in resistance at the electrode/electrolyte interface due to DNA damage. The results obtained via EIS correlate well with CV and SWV measurements. These results indicate that the size of the gold nanoparticles (8 and 30 nm) and their shell composition in our study did not have a significant impact on the amount of surviving DNA.

### 3.3. Effect of EtOH and a Physiological Concentration of NaCl on the DNA/GCE Biosensor

In the order to monitor the impact of various concentrations of EtOH on the surface-attached salmon sperm DNA, a set of CV measurements was conducted using a DNA/GCE biosensor with a physiological concentration of NaCl. The DNA/GCE biosensor was modified using 4 μL of 30%, 40%, or 50% *v*/*v* EtOH and 0.15 mol·L^−1^ NaCl. The modified DNA biosensor was irradiated for selected time intervals. The amount of surviving DNA was calculated based on the anodic peak current values measured by CV according to Equation (1) mentioned in the Section 2. Figure 4 shows the portion of surviving surface-attached DNA on the DNA/GCE biosensor in the presence/absence of selected additives (EtOH and NaCl) and under the simultaneous action of UV irradiation for the 20 to 900 s time interval. The presence of 30% (*v*/*v*) EtOH resulted in 94.7–56.0% of DNA maintenance, whereas under 40% (*v*/*v*) EtOH, dsDNA survival was between 93.2 and 65.5%, and with 50% (*v*/*v*) EtOH, 93.8–65.4% of dsDNA remained intact (Figure 4B). The results indicate the protective effect of 40% and 50% *v/v* EtOH for DNA against UV irradiation after 60, 300, 600, and 900 s of exposure. It is known that the addition of salt causes DNA structure compression [49]. The concentration of NaCl was the same in all experiments, so the protective effect was mainly due to the EtOH concentration. According to these results, we conclude that conformational changes caused by higher concentrations of EtOH can protect the surface-attached DNA layer from UV-induced damage.

### 3.4. Effect of Aggregated AuNPs on DNA/GCE in the Presence of EtOH and a Physiological Concentration of NaCl

**Figure 5:**
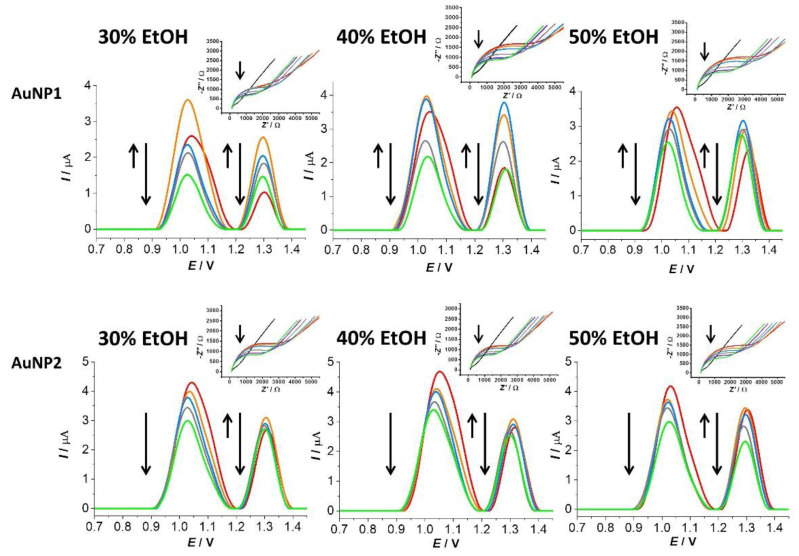
SWV responses of AuNP1 and AuNP2 impact on DNA on the DNA/GCE biosensor in the presence of 0.15 mol·L^−1^ NaCl and 30%, 40%, or 50% *v*/*v* EtOH after 0 s (red curve), 20 s (orange curve), 60 s (blue curve), 300 s (gray curve), and 900 s (green curve) of UV irradiation. The ↑ arrows represent opening of the DNA structure and the ↓ arrows represent DNA damage. The Nyquist plots show the response of the [Fe(CN)_6_]^3−/4−^ redox indicator on the DNA/GCE biosensor under the same experimental conditions. The black curve represents the response of bare GCE and the dark blue curve represents the response after 600 s of UV irradiation.

In the next step of the experiment, the effect of aggregated AuNP1 and AuNP2 on the surface-attached DNA was evaluated during simultaneous exposure to UV irradiation for 20–900 s time intervals using SWV, EIS (Figure 5), and CV, in which the amount of surviving DNA (Δ*I_rel_*) was calculated based on the anodic peak current values (Figure 4).

#### 3.4.1. Effect of Aggregated AuNP1 on DNA/GCE in the Presence of EtOH and a Physiological Concentration of NaCl

The Δ*I_rel_* values indicate a greater contribution of aggregated AuNP1 to DNA damage against dispersed AuNP1, especially in the presence of 30% *v*/*v* EtOH. With aggregated AuNP1, the amount of surviving DNA ranged from 88.4 to 52.3% (Figure 4C), and for dispersed AuNP1 in the presence of 30% *v*/*v* EtOH, the amount of surviving DNA ranged from 91.3 to 61.3% (Figure 4A), corresponding to the 20 to 900 s UV irradiation time interval. In the presence of 40% *v*/*v* EtOH, the Δ*I_rel_* values for aggregated AuNP1 ranged from 92.1 to 55.5% (Figure 4C). In the case of 50% *v*/*v* EtOH, there was no increase in DNA damage for aggregated compared to dispersed AuNP1, which also corresponds to Δ*I_rel_* values from 96.4 to 63.5% (Figure 4C). Nyquist diagrams obtained from EIS measurements show a decrease in the charge transfer resistance value across the electrode/electrolyte interface for the interaction of aggregated AuNP1. The EIS results confirm the CV measurements for all selected EtOH concentrations (inserts in Figure 5).

SW voltammograms correspond to the response of DNA bases guanine (1.04 V) and adenine (1.32 V) under the influence of aggregated AuNP1 and the simultaneous action of UV radiation in the given time interval. Figure 5 shows a shift of the guanine response to more negative potential at 20 s in the presence of 30% *v*/*v* EtOH due to the opening of the DNA double-helix structure (orange curve). The adenine residue response shows a visible increase in the current values while maintaining a stable potential during exposure to UV irradiation. Carnerero et al. reported that aggregated AuNPs remained preferentially located along the DNA double helix [50]. In our work, the decrease in the response of DNA base residues in the presence of aggregated AuNP1 and 30% *v/v* EtOH (Figure 5, red curve) supports this conclusion. The infiltration of the aggregated AuNP1 into the DNA structure corresponds to a potential shift of the guanine residue to more negative values. The increase in the current response of adenine residue is probably due to the sodium cation presence in the medium. The binding of alkali metal cations (such as Na^+^ or K^+^) to DNA bases was previously described. The strongest binding occurred within the adenine nucleotide, specifically at position N7 in its structure, which led to the breakdown of hydrogen bonds between adenine and thymine [51].

In the case of aggregated AuNP1 in the presence of 40% *v*/*v* EtOH, a similar interaction with the DNA can be observed as in the case of 30% *v*/*v* EtOH by SWV. A guanine residue potential shifted to negative values at 20 s of UV irradiation (orange curve), then remained stable with increasing UV irradiation time. The increase in the current response of the adenine residue up to 60 s of UV irradiation (blue curve) is due to the slower opening of the DNA double-helix structure. Xiaogang Han et al. reported that as the concentration of EtOH increases, smaller aggregates are formed [52]. When aggregated AuNP1s are smaller in the presence of 40% *v/v* EtOH, as in presence of 30% *v/v* EtOH, they are more easily incorporated into the DNA structure because the access of sodium cations to the adenine structure is better, and there is a significant increase in the current signal of adenine residues (Figure 5).

In the presence of 50% *v*/*v* EtOH, a different mechanism of interaction of aggregated AuNP1 with surface-bound DNA was observed than in the case of aggregates formed at lower concentrations of EtOH. SWV shows a potential shift of the signal of the residues of both the guanine adenine residue bases towards negative values after UV irradiation from 20 to 900 s (Figure 5). Another difference is the potential shift of the guanine residue, which does not remain stable after 20 s of exposure to UV radiation (orange curve); on the contrary, with increasing irradiation time, a further shift to negative potential values occurs (blue, gray, and green curves), indicating an increasing electrostatic interaction between aggregated AuNP1 and DNA. Electrostatic interaction affects the overall structure of DNA [53,54], which is why potential changes occur in the SWV response of both DNA base residues. Cheng et al. reported that Na^+^ preferentially interacts with the phosphate group of the DNA backbone [54]. Therefore, we considered that in the presence of 50% *v/v* EtOH, a weak electrostatic interaction probably occurred between aggregated AuNP1 and PO_4_^3−^. Incorporation into the DNA structure is also possible due to the small size of the aggregates formed in the presence of 50% *v/v* EtOH.

#### 3.4.2. Effect of Aggregated AuNP2 on DNA/GCE in the Presence of EtOH and a Physiological Concentration of NaCl

By evaluating the measured CV data, the amount of survived DNA was calculated in the presence of aggregated AuNP2 and a physiological concentration of NaCl. UV irradiation of the DNA/GCE biosensor occurred in the 20 to 900 s time interval. In the presence of 30, 40, and 50% *v*/*v* EtOH, Δ*I_rel_* ranged from 93.5 to 62.5%, 96.5 to 65.4%, and 79.1 to 57.1%, respectively (Figure 4D). In the case of aggregated AuNP2 in the presence of 30 and 40% *v*/*v* EtOH, the DNA damage did not increase under the given experimental conditions, as shown in Figure 4D. The Δ*I_rel_* for these two types of aggregates exceeded the percentage values of the amount of surviving DNA for the DNA/GCE biosensor itself. The most significant contribution to DNA damage was observed in the case of aggregated AuNP2 in the presence of 50% *v*/*v* EtOH under simultaneous UV irradiation, with the most significant damage after 20 and 60 s. A significant change in spectral properties that represent the different morphologies of these aggregates may also be reflected in this case. The Nyquist plots obtained by EIS also correlate well with the CV results (Figure 5, inserts).

SWV records show only a small shift of the potential for both DNA bases towards negative values in the presence of aggregated AuNP2 with 30 or 40% *v*/*v* EtOH (Figure 5), indicating an electrostatic interaction [43,44]. Intercalation is unlikely due to the larger dimensions of the aggregates. Opening of the DNA structure through an increase in the signal of the guanine residue did not occur at any EtOH concentration. The current response of adenine residue increased after the 20 s (orange curve), with a subsequent decrease in the signal with ascending UV irradiation time (60–900 s) (Figure 5). In the case of aggregated AuNP2 in the presence of 50% *v/v* EtOH, a shift of the guanine residue potential to more negative values was recorded after 20 s (orange curve), remaining stable with increasing UV irradiation duration. The potential shift of the adenine residue exhibits the same trend. We observed a more significant decrease in the current values of the adenine residue with increasing time of exposure to UV irradiation, as in the case of aggregated AuNP2 in environments with 30 and 40% *v*/*v* EtOH. The behavior of aggregated AuNP2 in the presence of 50% *v/v* EtOH is very similar to that of aggregated AuNP1 in the presence of 30% *v/v* EtOH.

## 4. Conclusions

The ethanol and NaCl-induced gold nanoparticle aggregation toxicity toward dsDNA was investigated with a DNA/GCE biosensor under simultaneous UV irradiation. The interaction of two sizes AuNP, namely AuNP1 (8 nm) and AuNP2 (30 nm), in the presence of 30, 40, or 50% *v*/*v* EtOH and 0.15 mol·L^−1^ NaCl with surface-attached DNA was studied. Due to the presence of salt and EtOH, the spontaneous aggregation of gold nanoparticles occurred in both cases. The aggregation was detected by UV-vis spectroscopy. The dispersed AuNP1 and AuNP2 did not affect the integrity of surface-attached DNA. The presence of NaCl and EtOH had a minimally protective effect on the DNA double helix against UV irradiation at higher concentrations of EtOH and with longer durations of irradiation due to the EtOH-induced structural changes of the double helix from the B form to the more condensed C form. Among all aggregates, the aggregated AuNP1 in the presence of 30% *v*/*v* EtOH contributed to DNA damage most significantly. The aggregated AuNP2 in the presence of 50% *v/v* EtOH showed similar behavior, but the contribution to DNA damage was less significant. The 93.5–60.0% surviving DNA after 20–900 s of UV irradiation of the DNA/GCE biosensor represents the limitation of the possible DNA damage/protection detection ranging from 93.5/6.5% to 60/40%. This DNA/GCE biosensor, together with simultaneous UV irradiation, provides a new signal detection strategy to assess not only DNA damage but also the protective effect of various compounds for DNA structure, with a broad range of potential applications.

## Data Availability

Not applicable.
